# Prognostic Role of Lymphocyte-to-Monocyte Ratio (LMR) in Patients with Intermediate-Stage Hepatocellular Carcinoma (HCC) Undergoing Chemoembolizations (DEM-TACE or cTACE) of the Liver: Exploring the Link between Tumor Microenvironment and Interventional Radiology

**DOI:** 10.3390/diseases12070137

**Published:** 2024-06-27

**Authors:** Roberto Minici, Massimo Venturini, Giuseppe Guzzardi, Federico Fontana, Andrea Coppola, Filippo Piacentino, Federico Torre, Marco Spinetta, Pietro Maglio, Pasquale Guerriero, Michele Ammendola, MGJR Research Team, Luca Brunese, Domenico Laganà

**Affiliations:** 1 Radiology Unit, University Hospital Dulbecco, 88100 Catanzaro, Italy; domenico.lagana@unicz.it; 2 Diagnostic and Interventional Radiology Unit, ASST Settelaghi, Insubria University, 21100 Varese, Italy; massimo.venturini@uninsubria.it (M.V.); federico.fontana@uninsubria.it (F.F.); andrea.coppola@asst-settelaghi.it (A.C.); filippo.piacentino@asst-settelaghi.it (F.P.); 3 Imagerie Vasculaire et Interventionnelle, Centre Hospitalier Princesse Grace, 98000 Monaco, Monaco; giuseppe.guzzardi@chpg.mc (G.G.); federico.torre@chpg.mc (F.T.); 4 Radiology Unit, Maggiore della Carità University Hospital, 28100 Novara, Italy; marco.spinetta@maggioreosp.novara.it; 5 Pain Management Unit, University Hospital Dulbecco, 88100 Catanzaro, Italy; pmaglio@aocz.it; 6 Department of Medicine and Health Sciences, University of Molise, 86100 Campobasso, Italy; pasquale.guerriero@unimol.it (P.G.); luca.brunese@unimol.it (L.B.); 7 Digestive Surgery Unit, University Hospital Dulbecco, 88100 Catanzaro, Italy; michele.ammendola@unicz.it

**Keywords:** inflammation-based scores, lymphocyte-to-monocyte ratio (LMR), hepatocellular carcinoma (HCC), transcatheter arterial chemoembolization (TACE), tumor microenvironment, biomarkers, cancer, drug-eluting microspheres (DEM), drug-eluting beads (DEB), precision medicine

## Abstract

Inflammation-based scores are biomarkers of the crosstalk between the tumor microenvironment and the immune response. Investigating the intricate relationship between the tumor stromal microenvironment, biomarkers, and the response to transcatheter arterial chemoembolization (TACE) is essential for early identification of TACE refractoriness or failure, providing insights into tumor biology and facilitating personalized therapeutic interventions. This study addresses a dearth of recent literature exploring the prognostic significance of the preoperative LMR in individuals from western countries diagnosed with stage B hepatocellular carcinoma (HCC) undergoing drug eluting microspheres TACE (DEM-TACE) or conventional TACE (cTACE). This international multi-center retrospective analysis included consecutive patients with stage B HCC who underwent TACE from January 2017 to June 2023. The study evaluated the ability of the preoperative LMR to predict complete response (CR), objective response (OR), sustained response duration (SRD) exceeding 6 months, successful downstaging at 6 months, progression-free survival (PFS) at 6 months, and overall survival (OS) at 6 months. The study population included 109 HCC patients and it was divided into low LMR (LMR < 2.24) and high LMR (LMR ≥ 2.24) groups, according to ROC curve analysis to select the optimal LMR cut-off value. High LMR was associated with lower Hepatitis C prevalence, higher absolute lymphocyte count, and a trend toward lower alpha-fetoprotein. The group with high LMRs exhibited superior CR rates (14.9% vs. 0%), overall OR (43.2% vs. 14.3%), and better PFS at 6 months (75.7% vs. 45.7%). The LMR, specifically categorized as <2.24 and ≥2.24, emerged as a robust predictor for treatment response and short-term outcomes in patients with stage B HCC undergoing DEM- or c-TACE.

## 1. Introduction

The third leading cause of cancer-associated mortality is represented by Hepatocellular Carcinoma (HCC). Annually, it accounts for 905,677 newly reported cases and contributes to 830,180 fatalities [1].

For patients with intermediate-stage HCC, or stage B disease as per the Barcelona Clinic Liver Cancer (BCLC) staging system, transcatheter arterial chemoembolization (TACE) is recommended as the primary therapeutic intervention, aligning with the EASL Guideline for HCC management [2,3,4,5]. Intermediate-stage HCC presents a diverse pathology, allowing for further subclassification, diverse therapeutic approaches, and variable outcomes [6,7]. As outlined in the 2022 BCLC strategy [5], TACE is not the sole treatment for stage B HCC, particularly in cases of diffuse, infiltrative, and extensive bilobar liver involvement. Moreover, successful downstaging opens the possibility for liver transplantation (LT) consideration in stage B patients initially beyond the Milan criteria [8]. Intriguingly, Yao et al. demonstrated that successful downstaging of HCC to within Milan criteria was associated with a low rate of HCC recurrence and excellent post-transplant survival, comparable to those meeting criteria at the outset [9].

Hence, the paradigm shift in the treatment options of stage B HCC will improve patient outcomes and it could allow surgical resection in resectable cases, downstaging therapy followed by curative treatments, transarterial radioembolization as an alternative or adjunct to TACE, or early systemic therapy in patients at high risk of TACE failure [10]. Therefore, it is crucial to comprehend the tumor’s biology to provide patients with tailored treatments. Multiple studies have indicated that the response to locoregional treatments, such as TACE, can serve as a surrogate marker for tumor biology [11,12,13]. Nevertheless, TACE refractoriness or failure emerges as a late-stage marker, given the customary quarterly imaging follow-up for patients undergoing locoregional therapies [14]. Any progression to advanced stage HCC is a critical event given the dismal outcome and that treatments for the advanced stage are still considered an unmet clinical need [10,15,16].

Conversely, inflammation-based scoring systems, such as the neutrophil-to-lymphocyte ratio (NLR), the lymphocyte-to-monocyte ratio (LMR), and the platelet-to-lymphocyte ratio (PLR), serve as indicators of the intricate interplay between the tumor stromal microenvironment and the immune response [17,18]. These scoring systems have been recommended as practical, easily and rapidly attainable, cost-effective, and reliable preoperative markers, demonstrating prognostic significance in patients with hepatocellular carcinoma (HCC) undergoing transcatheter arterial chemoembolization (TACE) [19]. They have been proposed as prognostic indicators for predicting recurrence, disease progression, and overall survival in HCC patients, as well as predictive factors for the response to TACE [20,21]. Investigating the intricate relationship between the tumor stromal microenvironment, biomarkers, and the response to TACE is essential for early identification of TACE refractoriness or failure, providing insights into tumor biology and facilitating personalized therapeutic interventions.

The prognostic utility of the LMR has been examined in various solid tumors [22,23], encompassing individuals with HCC [24,25]. Nevertheless, there is a dearth of recent literature exploring the prognostic significance of the preoperative LMR specifically in individuals from western countries diagnosed with stage B HCC undergoing drug eluting microspheres TACE (DEM-TACE) or conventional TACE (cTACE) [26]. This study aims to address this gap by evaluating the prognostic role of preoperative lymphocyte-to-monocyte ratio in patients with intermediate-stage HCC undergoing DEM-TACE. Specifically, we aim to evaluate the ability of the preoperative LMR to predict complete response (CR), objective response (OR), sustained response duration (SRD) exceeding 6 months, successful downstaging at 6 months, progression-free survival (PFS) at 6 months, and overall survival (OS) at 6 months.

## 2. Materials and Methods

### 2.1. Study Design

This study is an international, multi-center (Mater-Domini center of the Dulbecco University Hospital, Catanzaro, Italy; Circolo Hospital, Varese, Italy; Maggiore della Carità University Hospital, Novara, Italy; Centre Hospitalier Princesse Grace, Monaco, Principality of Monaco), retrospective analysis of prospectively collected data of consecutive patients with stage B HCC who had undergone, from January 2020 to December 2023, DEM-TACE or cTACE as the first-line treatment.

The inclusion criteria comprised the following: (I) DEM-TACE or cTACE for hepatocellular carcinoma in BCLC stage B [3,4,5]; (II) HCC diagnosed according to the European Association for the Study of the Liver criteria [2]; (III) Child–Pugh score up to 9; (IV) HCC not previously treated; (V) Eastern Cooperative Oncology Group performance status grade 0 [27]; and (VI) evaluation by a multidisciplinary team consisting of a hepatologist, liver surgeon, and interventional radiologist. Exclusion criteria were established as follows: (I) instances of missed radiological evaluations during the follow-up period; (II) serum creatinine levels surpassing 2.0 mg/dl; (III) platelet count falling below 50,000/μL and/or international normalized ratio (INR) exceeding 1.5; (IV) serum bilirubin levels equal to or exceeding 3 mg/dl; (V) contraindications for the administration of doxorubicin; (VI) previous chemoembolizations; (VII) high-flow arterioportal or arteriovenous shunts; (VIII) fever or other clinical and/or laboratory evidence of infectious or inflammatory status; and (IX) Child–Pugh score > B9.

Given the retrospective nature of the study, ethical committee approval was not deemed necessary. The research adhered to the ethical guidelines outlined in the Declaration of Helsinki. Prior to initiating the endovascular procedure, written informed consent was obtained from each individual participant.

### 2.2. Treatment

The chemoembolization procedure followed the technical details previously de-scribed in the literature [28,29], and its key characteristics are summarized here. Radial or femoral arterial access was established based on the operator’s preference (with more than 10 years of experience). Following the selective catheterization of the common hepatic artery using a 4 or 5 French diagnostic catheter, digital subtraction angiography (DSA) was performed. DSA was repeated from the proper hepatic artery, following its selective catheterization using a 2.7 French microcatheter (Progreat, Terumo, Japan). Tumor feeders were identified, and superselective catheterization was achieved using the microcatheter. Chemoembolization was carried out using PEG–based microspheres of 200 ± 50 micrometers, loaded with 75 mg of doxorubicin and mixed with iodinated contrast. Drug administration was halted when stasis was maintained for at least 10 cardiac beats [30]. cTACE was performed following the technique previously described in the Standard of Practice by the Cardiovascular and Interventional Radiological Society of Europe [28]. The TACE technique (DEM-TACE or cTACE) was chosen at the discretion of the operator. Each patient underwent clinical, laboratory, and imaging follow-up at 1 month and 3 months post-procedure, and subsequently every 3 months. Contrast-enhanced CT or gadolinium-enhanced MRI was employed for follow-up imaging.

### 2.3. Outcomes and Definitions

The primary outcome is the ability of preoperative LMR to predict PFS at 6 months. The ability of preoperative LMR to predict complete response (CR), objective response (OR), sustained response duration (SRD) exceeding 6 months, successful downstaging at 6 months, and overall survival (OS) at 6 months defined the secondary outcomes.

LMR was calculated as absolute lymphocyte count (number of lymphocytes/µL) divided by absolute monocyte count (number of monocytes/µL). The preoperative laboratory assessment was conducted no later than 48 hours before TACE. CR and OR were evaluated at 1-month imaging follow-up. SRD, PFS, and OS were evaluated at 6 months from the first TACE. Technical success was characterized by the successful delivery of the entire planned doxorubicin dose and the attainment of stop flow for at least 10 cardiac beats, according to the Cardiovascular and Interventional Radiological Society of Europe (CIRSE) standard of practice [28]. Evaluation of treatment response adhered to mRECIST guidelines [31]. Complete response denoted the absence of intratumoral arterial enhancement in all target lesions. Partial response (PR) was identified by a minimum 30% reduction in the sum of the diameters of viable (contrast-enhancing) target lesions. Progressive disease (PD) signified a minimum 20% increase in the sum of the diameters of viable (enhancing) target lesions, while stable disease (SD) encompassed cases not meeting criteria for partial response or progressive disease. Patients exhibiting new lesions, vascular invasion, and/or metastases were categorized as experiencing progressive disease. Following previously established definitions, disease control was computed as the sum of complete response, partial response, and stable disease [32,33]. Objective response included patients who have experienced CR or PR. Sustained response duration was defined as the duration between the date of achieving complete response, partial response, or stable disease and the date of progression.

### 2.4. Statistical Analysis

Data were recorded and organized in a Microsoft Excel spreadsheet (Microsoft Inc, Redmond, WA, USA), with subsequent statistical analyses conducted using SPSS software (SPSS, version 26 for Windows; SPSS Inc, Chicago, IL, USA). Our examination focused on the per-protocol population, comprising all randomly assigned patients who had undergone a chemoembolization procedure and completed imaging follow-up. To assess the normality assumption of the data, the Kolmogorov–Smirnov test and Shapiro–Wilk test were employed [34]. Categorical data are reported as frequency (percentage value) [35], while continuous, normally distributed data are presented as mean ± standard deviation [36]. Continuous data not following a normal distribution are expressed as median (interquartile range: 25th and 75th percentiles—IQR) [37]. Statistical differences for continuous, normally distributed data were evaluated using the unpaired Student’s *t*-test [38], whereas the Chi-squared or Fisher’s exact test [39] was applied for categorical data. The Mann–Whitney test was employed for continuous data not adhering to normal distribution. The cut-off value for the LMR was determined by using receiver operating characteristic (ROC) curve for 6-month PFS [40]. The ROC curve connects the coordinate points using “1—specificity (false positive rate)” as the x-axis and “sensitivity” as the y-axis for all cut-off values measured from the test results. The best area under the curve (AUC) defined the optimal cut-off point, thus maximizing sensitivity and specificity. Simple and multiple logistic regression analyses were conducted to evaluate potential factors predicting the occurrence of CR, OR, SRD > 6 months, downstaging, 6-month PFS, and 6-month OS [41]. An 80% subset of the dataset served as the training set, while the remaining 20% constituted the testing set. Variables exhibiting a significance level of *p* < 0.05 in univariable analyses were included in the multivariable logistic regression analyses [42].

## 3. Results

**Figure 1:**
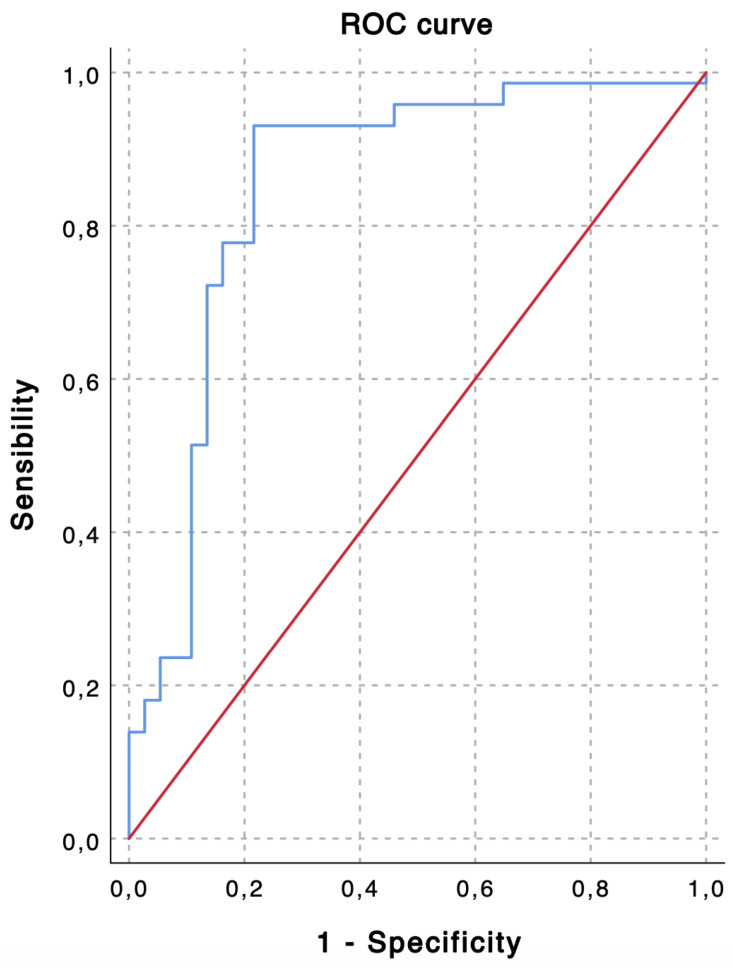
The receiver operating characteristic (ROC) curve shows predictive values of lymphocyte-to-monocyte ratio (LMR) for 6-month progression-free survival (PFS). An LMR value of 2.24 demonstrates the highest accuracy for the prediction of 6-month PFS (sensibility, 0.931; specificity, 0.784; area under the curve, 0.851).

TACE was performed as the first-line treatment in 109 consecutive patients with intermediate stage HCC. Based on the ROC curve analysis for the prediction of 6-month PFS (Figure 1), a cut-off value of 2.24 was chosen to divide the population into a low LMR group (Group 1—LMR < 2.24, n = 35, 32.1%) and a high LMR group (Group 1—LMR ≥ 2.24, n = 74, 67.9%). The overall performance of the ROC curve is defined by an area under the curve of 0.851, thus highlighting a good accuracy of the diagnostic test for the prediction of 6-month PFS [43].

Table 1 provides a comprehensive overview of the 109 patients with liver malignancies, stratified into Group 1 (low LMR, n = 35) and Group 2 (high LMR, n = 74). Noteworthy distinctions emerged, with Group 2 exhibiting a markedly lower prevalence of Hepatitis C compared to Group 1 (20% vs. 54.1%, *p* = 0.001). Group 2 also demonstrated a significantly higher LMR (4.92 vs. 2.82, *p* < 0.001). While age, sex distribution, and markers of liver function did not exhibit statistically significant differences, the neutrophil count (*p* = 0.011) and lymphocyte count (*p* < 0.001) were notably disparate between the two groups.

**Figure 2:**
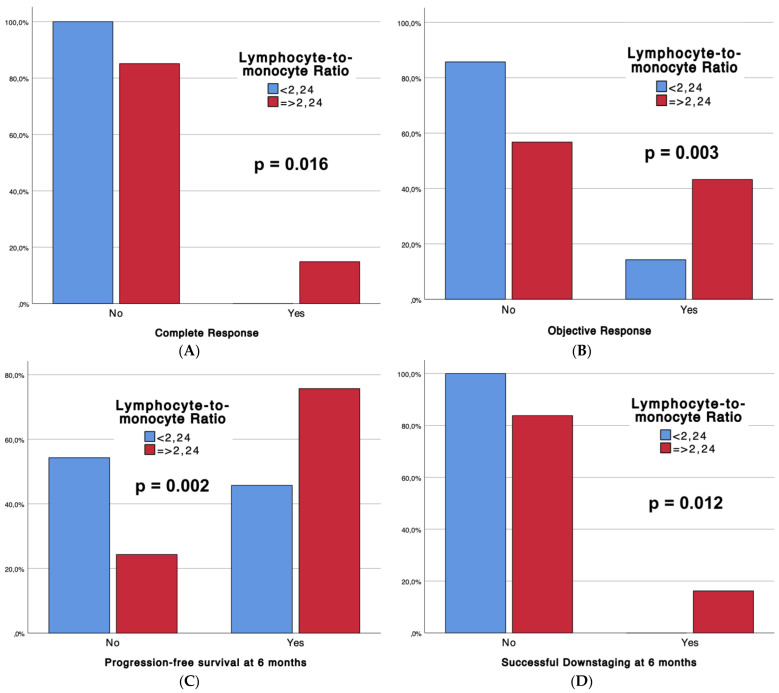
Boxplots representing complete response (**A**), objective response (**B**), progression-free survival at 6 months (**C**), and successful downstaging at 6 months (**D**), according to LMR Groups.

Table 2 outlines the outcomes of the interventional procedures, comparing patients with low LMRs (Group 1, n = 35) and high LMRs (Group 2, n = 74), with associated p-values. Technical success rates were universally high (100%) across both groups. Strikingly, significant differences emerged in tumor response, with Group 2 demonstrating superior complete response (CR) rates (14.9% vs. 0%, *p* = 0.016) and overall objective response (CR + partial response) (43.2% vs. 14.3%, *p* = 0.003). Moreover, Group 2 exhibited enhanced progression-free survival at 6 months (75.7% vs. 45.7%, *p* = 0.002) and a higher incidence of successful downstaging (16.2% vs. 0%, *p* = 0.012). Notably, adverse events (CTCAE classification) were more frequent in Group 2 (54.1% vs. 28.6%, *p* = 0.013). Furthermore, boxplots (Figure 2) are provided to depict significant differences in complete response, objective response, progression-free survival at 6 months, and sustained response duration ≥6 months, between LMR Groups.

Simple logistic regression analyses showed that age, Hepatitis C virus, α-fetoprotein, lymphocyte count, LMR and LMR groups (<2.24; ≥2.24), were significant single predictors of objective response occurrence. Multiple logistic regression analyses showed that age, Hepatitis C virus, α-fetoprotein, lymphocyte count, and LMR groups (<2.24; ≥2.24), were significant multiple predictors of objective response occurrence. It is noted that the group with an LMR of 2.24 or more is associated with an average increase of 1.578 in the log-odds of objective response occurrence. The percentage accuracy in the classification of the multiple binomial logistic regression model is 78.9%, meaning that 78.9% of objective response instances can be correctly classified with the independent variables added. Details are reported in Table 3.

Simple logistic regression analyses showed that neutrophil count, lymphocyte count, monocyte count, LMR and LMR groups (<2.24; ≥2.24), were significant single predictors of progression-free survival at 6 months. Multiple logistic regression analyses showed that monocyte count was the only significant predictor of progression-free survival at 6 months. A second multiple logistic regression analysis was conducted to exclude the possible interference effect of lymphocyte and monocyte counts with the LMR, thus maintaining the independence of the tested variables. Interestingly, LMR groups (<2.24; ≥2.24) were found to be a significant predictor of progression-free survival at 6 months. It is noted that the group with an LMR of 2.24 or more is associated with an average increase of 1.162 in the log-odds of progression-free survival at 6 months. The percentage accuracy in classification of the first multiple binomial logistic regression model is 85.3%, meaning that 85.3% of 6-month progression-free survival instances can be correctly classified with the independent variables added. Details are given in Table 4.

## 4. Discussion

Key insights derived from our investigation can be summarized as follows:

- Based on the ROC curve analysis, a cut-off value of 2.24 was chosen to divide the population into a low LMR group and a high LMR group. An area under the curve of 0.851 highlights good accuracy in predicting 6-month PFS;
- Strong correlations between the LMR and treatment response, progression-free survival, and successful downstaging at 6 months are noted;
- Hepatitis C virus infection, alpha-fetoprotein levels, lymphocyte count, and the LMR emerged as significant predictors affecting objective response occurrence and progression-free survival at 6 months. The LMR, categorized as <2.24 and ≥2.24, proved to be a robust predictor in both analyses (*p* = 0.001 and *p* < 0.001, respectively). These results underscore the prognostic role of the LMR in predicting treatment response and short-term outcomes in patients with stage B HCC undergoing TACE.

Inflammation-based scoring systems such as the NLR, LMR, and PLR offer practical and reliable markers for patients with solid cancers, including HCC [17,18]. These scores, reflecting the interplay between the tumor biology, the tumor stromal microenvironment (TME), and the immune response, serve as valuable prognostic indicators, predicting recurrence, disease progression, overall survival, and the response to TACE [20,21]. Exploring this intricate relationship is essential for early prediction of TACE refractoriness or failure, providing insights into tumor biology and enabling personalized therapeutic interventions [26]. The LMR has emerged as a prognostic marker in patients with solid tumors [22]. Nevertheless, the intricate mechanisms underlying how the LMR impacts prognosis remain elusive [44]. Lymphocytes, pivotal in immunosurveillance, contribute significantly to anticancer immune responses when infiltrating the TME, correlating with enhanced survival [45,46]. Malignant cells hinder the proliferation of cytotoxic T lymphocytes (CTLs) within the tumor by generating immunosuppressive cytokines like interleukin (IL)-10, vascular endothelial growth factor (VEGF), and transforming growth factor beta (TGF-β). Additionally, they deplete IL-2, a crucial cytokine essential for sustaining CTL function [47]. Tumor-associated macrophages (TAMs), a subset of monocytes activated around tumors, exert influence on the metastatic process by modulating the TME, and their high infiltration is linked to an unfavorable prognosis [48,49]. The LMR, encompassing both lymphocytes and monocytes, emerges as a prognostic factor and surrogate marker of tumor-infiltrating lymphocytes (TILs) and TAMs [44]. Inhibition of the PD-1/PD-L1 immune checkpoint pathway improves survival in cancer patients [50]. Notably, a significant correlation has been observed between low LMRs, heightened PD-L1 expression, and poor prognosis in HCC patients, suggesting an augmented presence of monocyte-derived cells in the HCC microenvironment thus resulting in cytokine-induced increased PD-L1 expression [51]. 

Despite investigating the LMR as a predictive and prognostic factor in HCC, studies are limited and highly heterogeneous, varying in terms of HCC stage, administered treatment, timing of pre- or post-operative inflammation-based score assessment, population sample size (often represented by a single ethnicity), statistical methods used to determine specific cut-off values, study design, and chosen endpoint. More specifically, previous reports have predominantly centered on Asian patients undergoing hepatectomy, transplantation, percutaneous ablations, cTACE, or TACE combined with percutaneous ablations. However, the literature lacks works specifically addressing stage B HCC patients from western countries undergoing TACE with lipiodol (cTACE), microspheres (DEM-TACE), or beads (DEB-TACE) [26]. Lin et al. retrospectively investigated the prognostic value of the preoperative LMR in HCC patients undergoing curative hepatectomy. The LMR was found to be an independent predictor of OS and RFS in HBV–associated HCC patients after surgery, with a defined optimal cutoff value of 3.23 [25]. Recently, Wang et al. examined the prognostic performance of the monocyte-to-lymphocyte ratio (MLR) at recurrence, advocating better prediction of tumor biological behavior. In their cohort of 606 HCC patients undergoing combined treatment with TACE and ablative therapy, they observed that the MLR is a good predictor of early relapse and survival, both when assessed preoperatively and after recurrence. Patients with a low MLR exhibited a higher median recurrence-free survival (RFS) than those with a high MLR (27.9 months vs. 17.8 months, *p* < 0.001) [52]. In a diverse group of 204 individuals diagnosed with HCC and treated with RFA plus TACE, individuals exhibiting an elevated LMR experienced extended OS compared to those with a lower LMR. Furthermore, the Cox proportional-hazards model recognized the LMR as a significant prognostic indicator for OS [53]. Furthermore, Lin et al. retrospectively analyzed 128 patients with intermediate-stage HCC who underwent TACE as their primary treatment, finding a significant contrast in PFS between the high LMR group and the low LMR group [54]. Interestingly, in a retrospective analysis involving 180 individuals, the combined presence of a low NLR at baseline along with a high LMR proved to be a reliable indicator for forecasting OS after TACE [55]. 

Interestingly, we found that absolute lymphocyte count (ALC) is a strong predictor of objective response and 6-month progression-free survival in a logistic regression analysis. Similarly, Lin et al. recorded a correlation between reduced ALC levels and OS, along with a state of lymphocytopenia in the group with low LMR [25]. These clinical findings are consistent with speculations on the biological plausibility of the phenomenon, including the ability of lymphocytes to infiltrate the TME in the form of TILs, thus playing a crucial role in the anti-tumor immune response, including HCC [56]. The LMR might possess an even stronger diagnostic value, considering that it takes into account not only the ALC but also the absolute monocyte count, thereby encompassing TAMs and their tumor-promoting effect [25,57]. To the best of our knowledge, this is the first study evaluating the prognostic role of the preoperative LMR in patients from western countries with intermediate-stage HCC undergoing DEM-TACE or cTACE. Chi-squared and Fisher’s exact tests demonstrated a positive correlation between LMR levels and response to TACE as well as between LMR levels and short-term outcomes (i.e., 6-month OS, PFS, and downstaging). Simple logistic regression analyses showed that an LMR ≥ 2.24 effectively predicts OR and 6-month PFS. On multivariate analysis, the LMR persisted as an independent predictor for both OR and 6-month PFS. Noteworthily, we included more patients within the Child–Pugh class B than previous reports, such as those by Shen et al. and Yang et al. (i.e., 61.4% vs. 36.8% vs. 5.1%, respectively) [53,58]. According to Itoh et al., HCC patients exhibiting a low preoperative LMR are associated with a more aggressive tumor behavior, characterized by larger tumor size and a higher serum AFP concentration, compared to patients with a high preoperative LMR [51]. Similarly, in our study, a trend toward higher AFP levels was observed in the low LMR group, further fueling speculation about the LMR’s potential to reflect the balance between the tumor and the host immune surveillance. Hence, the primary discovery of our study, aligning with the prior literature, is underscored by the evidence that preoperative LMR serves as a robust predictor of therapy response and short-term outcomes in patients with stage B HCC undergoing TACE. This finding substantiates the hypothesis regarding the role of the LMR as a biomarker reflecting the balance between immune response and tumor-promoting inflammation.

The interpretations of the present study’s results should be considered in light of potential limitations. Firstly, there was no formal direct examination of the tumor microenvironment. Secondly, the retrospective nature of the study introduced an inherent selection bias. Thirdly, the study relies on a small sample size with the evaluation of short-term outcomes only. Therefore, to determine the optimal LMR cut-off, we constructed a ROC curve based on 6-month PFS. We excluded the 6-month OVS and successful downstaging at 6 months due to their low representation in our study population. Furthermore, multiple hypothesis testing should be considered, given the use of both the Kolmogorov–Smirnov and Shapiro–Wilk tests. Multiple testing involves examining more than one hypothesis simultaneously. When numerous hypotheses are tested, each with a designated Type I error probability, the likelihood of committing at least one Type I error increases, often significantly, with the number of hypotheses. Lastly, while inflammation-based scores prove effective in predicting TACE response, PFS, and downstaging, it is crucial to avoid assuming that early aggressive therapies like systemic treatments or transarterial radioembolization can necessarily improve outcomes in patients with unfavorable inflammation-based scores. The practical utility of these scores as early markers of adverse tumor biology for selecting tailored therapeutics still requires validation through additional studies.

## 5. Conclusions

In conclusion, this is the first study evaluating the prognostic role of preoperative lymphocyte-to-monocyte ratio in patients from western countries with intermediate-stage HCC undergoing DEM-TACE or cTACE. Inflammation-based scores are biomarkers of the crosstalk between tumor biology, the TME, and the immune system. The lymphocyte-to-monocyte ratio is a practical, easily and rapidly attainable, cost-effective, and reliable preoperative marker, usable to strongly predict treatment response to TACE and short-term outcomes such as 6-month PFS in patients with intermediate-stage HCC. Future perspectives involve conducting studies that directly correlate the LMR, TAMs/TILs, and clinical outcomes. Additionally, large multicenter studies are needed to validate the clinical utility of LMRs as a prognostic marker that might contribute to the selection of the optimal tailored treatment option for each HCC patient in clinical practice.

## Figures and Tables

**Table 1:** Baseline demographic and clinical data.

Variables	All Patients (n = 109)			
		Group 1 Low LMR (n = 35)	Group 2 High LMR (n = 74)	*p* Value
Age (years)	57.1 (±13.7)	54.4 (±14.2)	58.4 (±13.4)	0.124
Sex (F)	32 (29.4%)	14 (40%)	18 (24.3%)	0.093
Hepatitis B virus	15 (13.8%)	4 (11.4%)	11 (14.9%)	0.627
Hepatitis C virus	47 (43.1%)	7 (20%)	40 (54.1%)	0.001
Non-alcoholic fatty liver disease	9 (8.3%)	5 (14.3%)	4 (5.4%)	0.116
Alcoholic liver disease	45 (41.3%)	18 (51.4%)	27 (36.5%)	0.139
α-Fetoprotein (ng/mL)	236.5 (±225.8)	311.9 (±286.7)	200.8 (±182.1)	0.110
Carbohydrate antigen 19-9 (U/mL)	11.4 (±13.6)	12.3 (±13.7)	10.9 (±13.6)	0.803
γ-Glutamyltransferase (U/L)	88.7 (±56.8)	91.8 (±71.5)	87.2 (±48.9)	0.953
Alkaline phosphatase (U/L)	52.4 (±21.9)	54.3 (±21)	51.5 (±22.4)	0.285
Aspartate transaminase (U/L)	64.1 (±28.7)	57.7 (±29.3)	67.1 (±28.1)	0.296
Alanine transaminase (U/L)	68.5 (±25.1)	70.3 (±25.8)	67.6 (±24.9)	0.422
Albumin (g/L)	30.1 (±2.7)	29.5 (±2.4)	30.4 (±2.8)	0.116
Total bilirubin (mg/dL)	1.12 (±0.4)	1.05 (±0.37)	1.15 (±0.42)	0.165
Prothrombin time (seconds prolonged)	6.4 (±1.4)	6.3 (±1.4)	6.5 (±1.4)	0.329
Ascites	0 (0%)	0 (0%)	0 (0%)	NA
Child–Pugh score, A6/B7/B8/B9	5 (4.6%)/37 (33.9%)/65 (59.6%)/2 (1.8%)	0 (0%)/13 (37.1%)/21 (60%)/1 (2.9%)	5 (6.8%)/24 (32.4%)/44 (59.5%)/1 (1.4%)	0.421
Cirrhosis	107 (98.2%)	34 (97.1%)	73 (98.6%)	0.584
Platelet count (No. ×103/μL)	135.1 (±51)	128.5 (±43.2)	138.2 (±54.3)	0.597
Hemoglobin (g/dL)	11.5 (±1.43)	11.8 (±1.48)	11.4 (±1.40)	0.241
White blood cell count (per μL)	4642 (±825.6)	4771 (±722.7)	4580 (±868)	0.136
Neutrophil count (per μL)	3332 (±776.2)	3604.9 (±747.9)	3203 (±760.7)	0.011
Lymphocyte count (per μL)	901.8 (±322.4)	710.8 (±287.6)	992.2 (±299.1)	<0.001
Monocyte count (per μL)	245.8 (±81.4)	277.1 (±79.2)	231.1 (±78.7)	0.003
Lymphocyte-to-monocyte ratio (LMR)	4.25 (±2.29)	2.82 (±1.58)	4.92 (±2.26)	<0.001
Number of tumors, 1/2/3	42 (38.5%)/34 (31.2%)/33 (30.3%)	12 (34.3%)/11 (31.4%)/12 (34.3%)	30 (40.5%)/23 (31.1%)/21 (28.4%)	0.773
Maximum tumour size (cm)	4.50 (±1.13)	4.42 (±1.10)	4.54 (±1.15)	0.651
Bilobar disease	46 (42.2%)	15 (42.9%)	31 (41.9%)	0.924
Capsule	56 (51.4%)	20 (57.1%)	36 (48.6%)	0.407

**Table 2:** Outcomes data.

Variables	All Patients (n = 109)			
		Group 1 Low LMR (n = 35)	Group 2 High LMR (n = 74)	*p* Value
Technical success	109 (100%)	35 (100%)	74 (100%)	NA
Tumour response				0.017
*CR*	11 (10.1%)	0 (0%)	11 (14.9%)	
*PR*	26 (23.9%)	5 (14.3%)	21 (28.4%)	
*SD*	49 (45%)	20 (57.1%)	29 (39.2%)	
*PD*	23 (21.1%)	10 (28.6%)	13 (17.6%)	
Complete response	11 (10.1%)	0 (0%)	11 (14.9%)	0.016
Objective response (CR + PR)	37 (33.9%)	5 (14.3%)	32 (43.2%)	0.003
Sustained response duration ≥ 6 months	57 (52.3%)	19 (54.3%)	38 (51.4%)	0.775
Overall survival at 6 months	109 (100%)	35 (100%)	74 (100%)	NA
Progression-free survival at 6 months	72 (66.1%)	16 (45.7%)	56 (75.7%)	0.002
Successful downstaging at 6 months	12 (11%)	0 (0%)	12 (16.2%)	0.012
Post-procedural clinical complications (CIRSE classification)	33 (30.3%)	12 (34.3%)	21 (28.4%)	0.340
*Grade 1*	28 (25.7%)	11 (31.4%)	17 (23%)	
*Grade 2*	0 (0%)	0 (0%)	0 (0%)	
*Grade 3*	5 (4.6%)	1 (2.9%)	4 (5.4%)	
*Grade* ≥ *4*	0 (0%)	0 (0%)	0 (0%)	
Adverse Events (CTCAE classification)	50 (45.9%)	10 (28.6%)	40 (54.1%)	0.013
*Grade 1*	29 (26.6%)	3 (8.6%)	26 (35.1%)	
*Grade 2*	16 (14.7%)	6 (17.1%)	10 (13.5%)	
*Grade 3*	5 (4.6%)	1 (2.9%)	4 (5.4%)	
*Grade 4*	0 (0%)	0 (0%)	0 (0%)	

**Table 3:** Logistic regression analysis (Simple—Multiple) of predictive factors affecting objective response occurrence.

Predictors	Coeff.	Std. Err.	Wald	*p* > |z|
Age (years)	0.530/0.071	0.02/0.02	9.093/8.653	0.003/0.003
Sex	−0.172	0.45	0.147	0.702
Hepatitis C virus	−0.860/−2.367	0.43/0.68	4.001/12.086	0.045–0.001
α-Fetoprotein (ng/mL)	−0.004/−0.004	<0.01/<0.01	7.178/4.833	0.007–0.028
Albumin (g/L)	0.047	0.74	0.404	0.525
White blood cell count (per μL)	0	0	0.112	0.738
Neutrophil count (per μL)	0	0	0.468	0.494
Lymphocyte count (per μL)	0.003/0.002	<0.01/<0.01	13.897/4.054	<0.001/0.044
Monocyte count (per μL)	−0.005	<0.01	3.274	0.070
Lymphocyte-to-monocyte ratio (LMR)	0.325/0.247	0.10/0.18	11.283/1.792	0.001/0.181
LMR Groups (<2.24; ≥2.24)	1.520/1.578	0.54/0.74	8.009/4.488	0.005–0.034

**Table 4:** Logistic regression analysis (Simple—Multiple I—Multiple II) of predictive factors affecting progression-free survival at 6 months.

Predictors	Coeff.	Std. Err.	Wald	*p* > |z|
Age (years)	−0.014	0.01	0.804	0.370
Sex	0.222	0.44	0.255	0.614
Hepatitis C virus	−0.174	0.41	0.182	0.669
α-Fetoprotein (ng/mL)	−0.002	<0.01	3.305	0.069
Albumin (g/L)	−0.009	0.07	0.014	0.906
White blood cell count (per μL)	0	0	2.344	0.126
Neutrophil count (per μL)	−0.001/0/0	0/0/0	4.629/0.202/2.393	0.031/0.653/0.122
Lymphocyte count (per μL)	0.003/0.003	<0.01/<0.01	15.125/3.431	<0.001/0.064
Monocyte count (per μL)	−0.017/−0.017	<0.01/<0.01	20.299/5.261	<0.001/0.022
Lymphocyte-to-monocyte ratio (LMR)	0.777/0.200	0.17/0.41	20.873/0.234	<0.001/0.629
LMR Groups (<2.24; ≥2.24)	1.307/0.326/1.162	0.43/0.58/0.45	9.058/0.319/6.798	0.003/0.572/0.009

## Data Availability

The data presented in this study are available upon request from the corresponding author. The data are not publicly available due to privacy issues.

## Abbreviations

AFP: alpha-fetoprotein; ALC: absolute lymphocyte count; AUC: area under the curve; BCLC: Barcelona-Clinic Liver Cancer; CR: complete response; cTACE: conventional TACE; CTL: cytotoxic T lymphocyte; DEB: drug-eluting beads; DEM: drug-eluting microspheres; HCC: hepatocellular carcinoma; IL: interleukin; LMR: lymphocyte-to-monocyte ratio; LRTs: locoregional treatments; MLR: monocyte-to-lymphocyte ratio; MRI: magnetic resonance imaging; NLR: neutrophil-to-lymphocyte ratio; OR: objective response; OS: overall survival; PD: progressive disease; PFS: progression-free survival; PLR: platelet-to-lymphocyte ratio; PR: partial response; RFA: radiofrequency ablation; RFS: recurrence-free survival; ROC: receiver operating characteristic; SD: stable disease; SRD: sustained response duration; TACE: transcatheter arterial chemoembolization; TAMs: tumor-associated macrophages; TGF-β: transforming growth factor-β; TILs: tumor-infiltrating lymphocytes; TME: tumor microenvironment; VEGF: vascular endothelial growth factor.
